# The distributive fairness of out-of-pocket healthcare expenditure in the Russian Federation

**DOI:** 10.1007/s10754-019-09268-9

**Published:** 2019-06-13

**Authors:** Pavitra Paul

**Affiliations:** 1 University of Eastern Finland, Kuopio, Finland; 2 Aix-Marseille School of Economics, Aix-Marseille Université, Marseille, France

**Keywords:** Distribution, Expenditure, Fairness, Out-of-pocket, Quantile, Socioeconomic, D39, D69, H49, H89, I14, I39

## Abstract

This article examines the effects of socioeconomic position and urban–rural settlement on the distribution of out-of-pocket expenditure (OPE) for health in the Russian Federation. Data comes from 2005 to 2016 waves of the Russian Longitudinal Monitoring Survey. Concentration index reflects changes in the distribution of OPE between the worse-off and the better-off Russians over a 12-year period. Finally, unconditional quantile regression—a recentred influence function approach estimates differential impacts of covariates along the distribution of OPE. OPE is concentrated amongst the better-off Russians in 2016. Urban settlements contribute to top end OPE distribution for the richest and town settlements, at the median for the richest and the poorest. Our model for the analysis is unique in the context of study population, as it marginalises the effect over the distributions of other covariates used in the model.

## Introduction

Following the disintegration of the United Soviet Socialist Republics, the Russian healthcare system underwent significant changes: decentralisation of management, introduction of a mandatory health insurance system and institutionalisation of user fees for certain healthcare services in addition to or instead of free-of-charge services (Popovich et al. 2011). The manifestation is an obvious, to varying degrees, form of private financing in which citizens pay out of pocket (OPE) for healthcare services that they receive from the public system (Gaal and McKee 2004). A source of revenue that has grown in importance is OPE, including both formal co-payments and informal, under-the-table payments (Preker et al. 2002). Often, the boundaries between free and chargeable health services are blurred (Rechel et al. 2013). Regions in the Russian Federation impose different payment schemes in which, that formal and informal payments may substitute for one another (Aarva et al. 2009). There has also been an implicit response taking the form of allowing capacity to wither and tolerating an increasing gap between population entitlement and capacity to provide healthcare services (Balabanova et al. 2012).

Deferred, deficient and denied consumption of needed healthcare services are inextricably linked with OPE (Balabanova et al. 2012; Eaddy et al. 2012; Wang et al. 2011; Karaca-Mandic et al. 2010). Salkever (1976) has conceptualised financial and physical barriers to healthcare consumption as determinants of access to healthcare services. The effect of education level (attainment) on OPE has also been reported (Mahumud et al. 2017; Okello and Njeru, 2015**)** in recent times. Molla et al. (2010) demonstrates that a 10% increase in uneducated members leads to a 11% decrease in household health expenditure, ceteris paribus. Geographical access may not be a major barrier to seeking care in the former Soviet Union (FSU) countries (Balabanova et al. 2012). Financial affordability is an enabling factor that determines health system’s coverage (Shengelia et al. 2003).

OPE occurs at point of services delivery and is regressive because people with low incomes pay proportionally more than do those with high incomes (Falkingham 2004; Waters et al. 2004; Wagstaff et al. 1999). OPE creates inequitable burdens that precipitate forgoing essential household spending or incurring debts (Himmelstein et al. 2009). For a similar level of OPE, the impoverishing effect of OPE is more intense and sustained on the worse-off group of population than it is on the better-off groups. Wagstaff and van Doorslaer (2003) have demonstrated the high-intensity effect of OPE on worse-off socioeconomic strata (SES) of the population relative to their better-off counterparts.

OPE for healthcare services generates utilisation inequalities and impoverishes women, and lower-income and socially marginalised groups (Taylor 2009). Further, the OPE effect is also associated with a poor health status (Eaddy et al. 2012; Tamblyn et al. 2001; Heisler et al. 2010; Soumerai et al. 1991). Such consequences of cost-sharing are most common among the worse-off (Tamblyn et al. 2001; Lesen et al. 2013; Schoen et al. 2010; Chernew et al. 2008). Thus, the consequences related to the accessibility of healthcare services can be enormous.

The levels of OPE in the Russian Federation are relatively high for informal payments but low for formal ones (Tragakes and Lessof 2003). The percentage of citizens with a high level of OPE (above 10% of household income)^1^ is highest in the Russian Federation; also, in absolute terms, the worse-off group (equivalent disposable income below 60% of the nation’s median income) of population in the Russian Federation are likely to have the highest OPE compared to France, Slovenia, Australia, the United States and Poland (Baird 2016). Despite universal^2^ coverage, better-off Russians consume more healthcare services than worse-off groups, although the latter’s health status is often worse (Popovich et al. 2011).

Although the impoverishing effect of OPE is well documented, the differential impact of socioeconomic position (SEP) and settlement of residence along the distribution of OPE have not yet been examined. This study addresses policy-relevant empirical questions, that is the effect of (1) changes in the distribution of SEP on OPE distribution and (2) the urban–rural segmentation of the population in the distribution of OPE. While addressing these distributional questions, we also examine the changes in the distribution of OPE between the worse-off and the better-off Russians over a 12-year period (2005–2016). Section 2 describes the data to which our model is applied. Section 2 also presents our analysis strategy that examines trend in OPE distribution and methodological approach to identify factors that influence OPE at different points of its distribution. Section 3 presents evidences from descriptive statistics and from application of econometric models. We discuss our empirical results on differential impacts of SEP and geography of residence in the distribution of OPE in Sect. 4. Section 5 concludes with breakthrough approach of our study and also, the limitations.

### An overview of the Russian health system

The Health system of the Russian Federation is a compulsory (non-competitive) insurance based model. The distribution of source of funding approximates 65%, 15% and 20% respectively from federal compulsory insurance fund, oblast (regional) budget and federal budget. The funding from the federal budget is primarily for regional programs of national importance and activities. Oblast is responsible for service production and delivery. Insurance fund is a combination of contributions from employers and employees, and pooled fund (risk adjusted) from federal compulsory insurance fund. Compulsory medical insurance guarantees (Article 41, Constitution of the Russian Federation) free primary, secondary and tertiary healthcare at the point of service consumption within the regional health system of the Russian Federation. However, the demand often exceeds service provision at the health facility level (polyclinics and hospitals) and also each health facility is limited with the fixed quantum (quota for the year)^3^ of money (payment for volume of services rendered) available from compulsory insurance fund for each year. Further, the regional ability to invest in health infrastructure development and often to meet the ongoing expenses at the health facility level are limited by the inherent flaws in the centralised tax collection system (Ragozin et al. 2013). Such constraints compel citizens to pay of their own at the point of service consumption if the citizen need/wants immediate service and/or health facility is compelled to meet the expenses by utilising the available capacity to the best possible extent. Health facility(s) in consultation with regional ministry of health determines tariffs of out-of-pocket payment for certain services provided by the respective health facility. Although the individual contribution to compulsory medical insurance fund is uniform, substantial differences exist for services on payment at health facility(s) across regions in the decentralised health system of the Russian Federation. Payment (unofficial) to the individual service provider (nurse and doctor) for receiving attention in regard to quality (as perceived by the individual citizen) and to prioritise (documenting clinical need of service provision) care is not uncommon. Often, gratitude to the individual service provider is also expressed with gift (cash and/or kind). Health facilities are paid by (1) capitated payment mechanisms for primary care and ambulatory care services, and (2) ‘pay for performance’^4^ for in-patient and day care services. Overall, 50% of the total money available to the health system goes for primary level healthcare services, nearly 44%, to secondary level medical care and the rest about 6% is spent on tertiary level healthcare services.].

## Data and methods

We used data from 12 waves (2005–2016) of the Russian Longitudinal Monitoring Survey (RLMS). The RLMS (http://www.cpc.unc.edu/rlms) is an ongoing longitudinal household survey of the Russian Federation, designed to be representative^5^ of the Russian population. The survey contains an array of information on the economic, social, demographic, and health characteristics of respondents, their households, and the environment where they live. The RLMS applies a multi-stage sampling method with precomputed cross-sectional post-stratification weights. These weights adjust not only for design factors, but also for deviations from census characteristics.^6^

Our data is a mix of cross-sectional and panel data (in which a segment of the households were followed over time), although the panel is not a balanced one. 172,332 adult respondents nested in 79,480 households were enrolled in the study (Table 1).

**Table 1:** Data used

Survey year	Number of respondents (age ≥ 16)	Year-on-year attrition (%)
2005	9964	5.79
2006	12,082	23.95
2007	11,939	8.95
2008	11,537	7.42
2009	11,489	5.40
2010	17,363	37.06
2011	17,843	15.97
2012	18,183	14.23
2013	17,516	9.83
2014	14,786	5.36
2015	14,717	5.89
2016	14,913	5.14
Total	172,332	12.79

OPE included both formal and informal payments and the amount spent on gifts for care while seeking outpatient (ambulatory care) services, including diagnostic services from public-sector health facilities. Payment for medicines was not included in OPE. Age, level of education (three levels: incomplete secondary school, completed secondary school and higher education including vocational training), household size and settlement of residence were used as other predictor variables. Dummy variables for three different levels of education (attainment) captured the relationship with the pattern of service consumption and examined association with OPE. We used Kruskal–Wallis H test to determine if there were statistically significant differences between respondents of different levels of education on OPE. Our data did not have any respondent who had not visited ever any doctor (a proxy for service consumption) during the study period. Although standard deviation measures variability, we examined the skewness of OPE distribution for different SEPs and for different settlements by observing the difference between mean and median value of OPE for the respective group(s).

SES is a multifaceted concept: no direct measure is available. Heterogeneity in relevant individual and household circumstances, inter-temporal consumption smoothing, and inter-personal income sharing mean that neither measured current income^7^ nor consumption^8^ are particularly good proxies for capturing measures of economic welfare. To measure SES, studies have used variables, such as agricultural land ownership (Filmer and Pritchett 2001), farm animals and ownership of the place of residence (Schellenberg et al. 2003), and crowding (Cortinovis et al. 1993). Houweling et al. (2003) has found that inclusion of a sanitation facility variable increased inequality amongst households. Lindelow (2002) has demonstrated that the inclusion of infrastructure variables increased SES inequality in healthcare service use. We constructed the SES index by applying polychoric principal component analysis. Such an approach in constructing SES indices has enabled us to overcome the limitations of (1) income-based approaches (McKenzie 2003; Montgomery et al. 2000) and also (2) consumption-based approaches (Filmer and Pritchett 2001).

Principal component analysis works through the covariance or correlation matrix to extract the directions in the multivariate space that are the ‘most informative’, that is reflection of the greatest variability. Kolenikov and Angeles (2009) demonstrated that the proportion of explained variance estimated using the polychoric PCA method^9^ is more accurate than that generated using other methods. The polychoric procedure uses discrete variables and calculates what their correlation would be if they were on a continuous scale (Uebersax 2006).

On iteration of the principal factors, we found three variables, namely, living space in square metres, provision and type of central heating, and ownership of tractor, with loadings at 64%, 87% and 64% respectively. We used the weighted sum of standardised variables (living space in square metres, provision and type of central heating, and ownership of tractor) to obtain the SES score. Lastly, individuals were grouped into quintiles of different SEPs. With orthogonal rotation (varimax^10^), the value of the first factor was 0.9470; the second factor, 0.3213. The inclusion of a sufficiently broad range of variables and continuous variables as well enabled us to construct the SEP indices without problems of truncation (McKenzie 2003).^11^ Kaiser–Meyer–Olkin (measure of sampling adequacy) score was 0.87 and Bartlett test of sphericity indicated variables were not intercorrelated (determinant of the correlation matrix, 0.014 with *p* value = 0.000).

### Analysis strategy

Our measure of inequality in OPE was the concentration index (CI). A CI ranks individuals by SEP corresponding to OPE. A CI is sensitive to the distribution of population across SES and reflects the experience of the entire population, not just two extreme groups. The CI is equal to the estimation of $\beta$ (Kakwani et al. 1997) in Eq. 1. We applied a regression-based approach for the indirectly standardised CI. We used Newey–West regression estimator that corrects for autocorrelation, as well as any heteroskedasticity.

1 $$ 2\sigma_{R}^{2} \left[ {\frac{{E_{i} }}{\mu }} \right] = \alpha + \beta R_{i} + \mathop \sum \limits_{j} \delta_{j} x_{ji} + \mu_{i} , $$

where $\sigma_{R}^{2}$ is the variance of $R_{i}$, the $i$th individuals’ SEP. E is OPE (outcome variable) whose inequality is being measured and $\mu$ is its mean; $x_{j}$ are confounding variables, namely, age, level of education, household size and settlement of residence. Thus, CI is the slope of a line passing through the heads of a parade of people ranked by their SEP and their height proportional to the value of their OPE, expressed as a fraction of the mean. The CI taking a negative value indicates a concentration of OPE among the better-off.

Decomposition of the index is then performed using a two-step procedure: first, computing the ‘Recentred influence function (RIF)’ of the rank-dependent index (CI) and estimating density at that point using kernel methods, and then regressing the RIF on a set of covariates yielding the marginal effects of the covariates on the index. Our model was unconditional quantile regression (UQR) based on RIF.^12^ UQR frameworks were used for understanding differential impacts of covariates along the distribution of OPE.

This RIF is derived from ‘influence function (IF)’, which can be used to estimate the effect (or ‘influence’) of removing/adding an observation on the value of $v$ (any distributional function), where $v(F)$ is the statistic of interest calculated for distribution $F(y)$ and is defined (Hampel et al. 2005) as ${\text{IF}}\left( {{\text{y}};{\text{v}}\left( {\text{F}} \right)} \right) = {\text{Lim}}_{{\upvarepsilon \to 0}} \frac{{\left[ {{\text{v}}\left( {\left( {1 -\upvarepsilon} \right){\text{F}} +\upvarepsilon \cdot\updelta_{\text{y}} } \right) - {\text{v}}\left( {\text{F}} \right)} \right]}}{\upvarepsilon}$, $0 \le\upvarepsilon \le 1$, where ${\text{F}}$ represents the cumulative distribution for $y$ (outcome of interest, that is, OPE) and $\delta_{y}$ is a distribution that puts the population at the value of $y$. Thus, a RIF is obtained from IF as ${\text{RIF}}\left( {{\text{y}};{\text{v}}} \right) = {\text{v}}\left( {\text{F}} \right) + {\text{IF}}({\text{y}};{\text{v}})$. RIF regressions (Firpo et al. 2009) relate some distributional statistics $v$(F) to explanatory variables, x, that is, $v\left( F \right) = E_{x} (E[RIF(y;v,F) \big|x)])$. So, a regression-based model (Firpo et al. 2009) is ${\text{E}}[{\text{RIF}}({\text{y}},{\text{v}},{\text{F}})|{\text{x}})] =\upalpha +\upbeta{\text{x}}$. This method evaluates the impact of covariate(s) on selected distribution statistics. Most interestingly, this procedure is valid for distribution quantiles of y. Regression coefficients ($\beta )$ revealed how much the average influence of observations had varied with x, holding other covariates constant for given quantile. It also revealed how much the quantile would respond to a change in the distribution of x in the population when distribution of other covariates remains constant. RIF regression allowed changes in the distribution of x to affect the distribution of y, and this makes $v$(F).^13^

For the OPE (statistic of interest, that is, ${\text{y}}$) of any quantile τ, RIF $({\text{y}};Q_{\tau } ) = Q_{\tau } + \frac{{\tau - I\{ {\text{y}} \le Q_{\tau } \} }}{{f_{Y} (Q_{\tau } )}}$, where $Q_{\tau }$ refers to the τth quantile of unconditional distribution of ${\text{y}}$, $f_{Y} (Q_{\tau } )$ is the probability density function of ${\text{y}}$ evaluated at $Q_{\tau }$, and $I\{ {\text{y}} \le Q_{\tau } \}$ is an indicator variable to denote whether an outcome value is less than or equal to $Q_{\tau }$ or not. Thus, our regression model (Eq. 2) accounted for the possibility of differential impact that covariate(s) might have across various segments of the OPE distribution.

2 $$ {\text{E}}[{\text{RIF}}({\text{y}},Q_{\tau } )|{\text{x}})] = {\text{x}}_{{\gamma_{\tau } }} $$

$\gamma_{\tau }$ shows the unconditional quantile partial effects (UQPE), that is, the regression parameter of ${\text{RIF}}({\text{y}},Q_{\tau } )$ on ${\text{x}}$. UQPE measures the effect of an explanatory covariate on OPE (the outcome of interest) at the specific quantile (Firpo et al. 2009). Here, regression coefficient interpretation is direct, and linear approximation is valid only for marginal changes in x.

Finally, we decompose (Eq. 3) the inequality measure into a function of its (potential) causes.

3 $$ Q_{\tau } = {\text{E}}\left( {{\text{RIF}}\left( {{\text{y}},Q_{\tau } } \right)} \right) = {\text{E}}_{\text{X}} \left[ {{\text{E}}\left( {{\text{RIF}}\left( {{\text{y}},{\text{Q}}_{\tau } } \right)|x} \right)} \right] = {\text{E}}[{\text{x}}]\gamma_{\tau } . $$

The difference between groups at the τth quantile of OPE is

$$ \hat{\Delta }_{0}^{\uptau} = {\bar{\text{x}}}_{\text{B}} \left( {\hat{\gamma }_{{{\text{B}},\uptau}} - \hat{\gamma }_{{{\text{A}},\uptau}} } \right) + \left( {{\bar{\text{x}}}_{\text{B}} - {\bar{\text{x}}}_{\text{A}} } \right)\hat{\gamma }_{{{\text{A}},\uptau}} = \hat{\Delta }_{\text{S}}^{\uptau} + \hat{\Delta }_{\text{x}}^{\uptau} , $$

where ${\text{A and B}}$ are two different groups, $\hat{\Delta }_{\text{S}}^{\uptau}$ is the structural effect (explained by group differences in the coefficients), and $\hat{\Delta }_{\text{x}}^{\uptau}$ is the composition effect (explained by group difference in the distribution of characteristics). Therefore, the sum of contribution of each covariate is

$$ \hat{\Delta }_{\text{x}}^{\uptau} = \mathop \sum \limits_{{{\text{k}} = 1}}^{\text{K}} \left( {{\bar{\text{x}}}_{\text{Bk}} - {\bar{\text{x}}}_{\text{Ak}} } \right)\hat{\gamma }_{{{\text{Ak}},\uptau}} , $$

where $\left( {{\bar{\text{x}}}_{\text{Bk}} - {\bar{\text{x}}}_{\text{Ak}} } \right)\hat{\upgamma }_{{{\text{Ak}},\uptau}}$ is the contribution of the ${\text{k}}$th covariate to composition effect at $\uptau$ quantile (Firpo et al. 2009).

## Results

Table 2 presents descriptive statistics for 6 years, that is, three blocks with two intervals: 4 years and 6 years. The choice of intervals was guided by the year-on-year attrition percentage (Table 1). The representation of respondents by age, gender and settlement of residence was consistent in our study. The respondents from city and town population who were employed and had a higher level of education grew between 2005 and 2016. The result of test of independence showed that incomplete secondary school level education did not have statistically significant visit frequency to a doctor but visit frequency(s) to a doctor was statistically significant for respondents with completed secondary school and respondents with higher education.

**Table 2:** Descriptive statistics

	2016	2015	2010	2009	2006	2005
	Female (%)	Female (%)	Female (%)	Female (%)	Female (%)	Female (%)
*Agegroup*						
16–30 years	51.69	51.23	53.38	53.24	52.22	51.37
31–44 years	53.64	53.77	54.43	54.56	53.85	53.47
45–60 years	58.32	58.28	57.32	57.35	57.93	58.45
61–74 years	65.35	65.46	65.99	67.23	66.57	66.18
75 years +	73.88	74.34	75.12	75.47	76.46	77.51
*Settlement (%)*						
Oblast City	41.85	41.53	41.11	41.37	41.47	40.77
Town	26.93	26.29	26.18	25.9	26.43	25.91
Urban settlements	6.66	6.9	6.39	6.51	5.91	6.63
Rural	24.56	25.28	26.32	26.22	26.19	26.69
*Education (%)*						
Incomplete secondary school	17.03	17.77	20.05	21.62	23.78	21.14
Completed secondary school	29.78	29.85	33.1	29.87	33.65	35.32
Higher education, incl. vocational training	53.18	52.38	46.85	48.51	42.57	43.54
Employed (%)	55.77	55.75	58.3	57.86	56.01	54.36
Inflation-adjusted (Jan. 2005) income (per annum)	€	€	€	€	€	€
Mean	8106.91	7023.51	5908.65	4928.4	3382.74	2504.1
Median	6481.13	5903.86	4756.23	3669.18	2591.42	1800
Living space (mean m^2^)	40.11	39.79	38.47	37.67	36.02	36.15
Household size (mean)	3.36 (1.77)	3.35 (1.72)	3.35 (1.60)	3.28 (1.60)	3.32 (1.58)	3.33 (1.61)
Land ownership (%)	52.02	51.82	54.12	55.36	56.08	57.13
*Visit frequency to a doctor (%)*						
Less than once a year	29.71	33.0	31.84	31.66	39.01	37.57
At least once a year	59.1	56.47	57.48	56.74	50.21	51.8
At least once a month	11.19	10.53	10.68	11.6	10.78	10.63
Cramer’s V (association with paid for outpatient visit)	0.41	0.41	0.42	0.41	0.42	0.42
*Perceived health status (%)*						
Very good	1.98	1.7	1.66	2.23	1.63	1.73
Good	35.99	35.36	32.01	29.47	28.39	30.97
Average	49.6	50.42	53.09	53.79	55.39	52.96
Bad	11.02	10.95	11.63	12.84	12.65	12.16
Very bad	1.41	1.56	1.6	1.68	1.94	2.18
Paid for outpatient visit (%)	14.64	12.94	15.75	14.55	17.7	16.79
OPE	€	€	€	€	€	€
Mean	84.78	50.53	82.02	46.98	27.53	26.54
Median	31.8	18.59	24.5	23.17	14.42	8.84
*SES distribution (quintile %)*						
Poorest	19.9	19.48	18.8	19.85	18.88	19.92
Second-poorest	19.9	20.35	21.18	20.14	20.93	19.68
Middle	19.76	20.03	19.32	19.36	19.37	20.38
Second-richest	16.62	16.78	20.66	19.32	18.75	18.66
Richest	23.82	23.36	20.03	21.34	22.07	21.36
Inflation adjusted (Jan. 2005) mean Income (per annum)	€	€	€	€	€	€
Poorest	619.7	492.78	903.63	831.24	744.32	722.67
Richest	919.7	739.89	1500.68	1387.61	1474.56	1369.16
OPE (mean) by SEP	€	€	€	€	€	€
Poorest	1.32 (23.19)	0.25 (3.09)	1.43 (24.48)	0.65 (8.61)	0.50 (4.60)	0.78 (10.66)
2nd poorest	0.87 (14.87)	0.64 (6.36)	1.48 (16.30)	0.62 (6.52)	1.09 (9.50)	0.49 (4.59)
Middle	2.11 (27.38)	1.70 27.84)	3.91 (65.05)	1.68 (21.18)	1.23 17.20)	1.02 (9.92)
2nd richest	2.12 (25.49)	1.39 19.84)	4.37 (96.87)	1.07 (15.09)	1.42 14.40)	1.10 (11.05)
Richest	2.70 (45.44)	0.84 (9.75)	2.16 (22.93)	0.78 (8.37)	1.37 (9.50)	1.72 (25.29)
OPE (mean) by settlement	€	€	€	€	€	€
Oblast City	3.27 (43.88)	1.25 19.81)	4.27 (77.97)	1.56 (19.07)	1.79 15.25)	1.47 (19.41)
Town	0.84 (9.47)	0.92 15.23)	2.16 (37.45)	0.92 (11.94)	0.67 (5.55)	0.98 (10.02)
Small urban settlements	0.97 (11.49)	0.83 11.46)	2.03 (33.86)	0.59 (6.71)	1.26 19.71)	0.54 (4.28)
Rural	0.66 (9.59)	0.45 (6.84)	0.87 (13.88)	0.44 (5.98)	0.42 (4.61)	0.46 (8.02)
*Distribution OPE by settlement (% of respondents with OPE)*						
Oblast city	7.22	6.31	7.31	7.24	8.14	7.65
Town	3.56	3.24	4.2	3.82	4.41	4.14
Urban settlements	0.84	0.65	0.85	0.68	0.94	0.84
Rural	3.02	2.74	3.38	2.81	4.21	4.15
χ^2^	0.000	0.000	0.000	0.000	0.000	0.000
*Distribution OPE by SEP (% of respondents with OPE)*						
Poorest	2.35	1.69	2.21	2	2.65	2.87
Second-poorest	2.76	2.33	3.12	2.45	3.46	3.19
Middle	2.92	2.91	3.13	3.1	3.38	3.19
Second-richest	2.77	2.52	3.82	3.33	3.59	3.63
Richest	3.86	3.54	3.54	3.64	4.71	4.05
χ^2^	0.000	0.000	0.000	0.000	0.000	0.000
*Denial of service access attributable to financial affordability (% of respondents by SEP)*						
Poorest	0.74	0.91	0.89	0.76	1.98	1.85
Second-poorest	1.55	1.42	1.37	1.32	2.43	3.49
Middle	1.63	1.51	1.79	1.59	2.58	2.85
Second-richest	1.57	1.53	1.80	1.96	2.67	2.61
Richest	2.15	1.55	1.98	2.01	3.35	2.9
χ^2^	0.000	0.000	0.000	0.000	0.001	0.000
N	14,913	14,717	17,363	11,489	12,082	9964

Figures in the parentheses are standard deviation(s)

The average area of living space per household increased by 11% from 2005 to 2016, although the average size of the household remained almost the same over the period. The group who owned land was decreased by about 10% from 2005 to 2016. Inflation-adjusted income (wages earned) was more than three times larger in 2016 than in 2005, and the gap between mean and median income was reduced by 14% during the same period.

Compared with other years, going to the doctor less than once a year was at its lowest and, at the same time, at least once a year, at its highest in 2016. Notwithstanding same levels of reported ill health in the years 2005 and 2016, the proportion of respondents with below-average perceived health was 13% smaller in 2016 than in 2005. The year 2006 saw the largest percentage of respondents who paid for out-patient visits, but the average OPE was highest in 2016. The mean OPE peaked in 2016, and the gap between mean and median OPE was widest (almost three and half times) in 2010. Cramer’s V of 0.4 and above at each of the six time points over a 12-year period indicated a strong relationship between respondents ‘who paid for out-patient visits’ and visit frequency to a doctor.

There were 11.5% more respondents from the richest quintile in 2016 compared with 2005 (Table 2). Changes in SEP distribution of the population did not represent any consistent trend during the study period. Inflation-adjusted mean income was lowest for the poorest and the richest quintiles in 2015 and highest, in 2010 but the gap between the poorest and the richest was the smallest (48%) in 2016 and the highest (98%) in 2006.

The mean OPE increased substantially across SES between 2005 and 2016; however, no definite trend could be observed. Denial of services attributable to financial affordability was higher amongst the better-offs (Table 2). For the poorest, real income decreased by 14.25% between 2016 and 2005, but mean OPE increased by 69% during the same period, whereas, for the richest, real income decreased by 32.83% and mean OPE increased by 57% during that period (Table 2).

The test of independence between OPE and settlement of residence and between OPE and SEP implied the existence of an association of SEP and settlement of residence, and with OPE. Although it was difficult to infer any definite trend, the percentage of respondents with OPE was always relatively higher in urban (oblast city and town) settlements and relatively less in rural (small urban settlement and village) settlements across all the years. Table 2 also reflected the better-offs (the second-richest and richest groups combined) with OPE were greater in number compared with the worse-offs (the second-poorest and the poorest groups combined) during all the years. An overall consistent trend of association between SEP and denial of service access attributable to financial affordability was reflected among respondents in the study (Table 2).

Table 3 shows the concentration index for OPE and distribution effect induced by different explanatory variables from 2005 to 2016. Although the C1 was positive throughout the study period, there were variations in OPE inequality. Seen from a longer time perspective, CI decreased by 20% from 2005 to 2016, implying a shift towards higher OPE for the better-off group of population. The contribution of different explanatory variables was not consistent over the study period. A negative contribution indicates a concentration of corresponding factors among the worse-off individuals, thus lowering the risk of higher OPE for the worse-offs. Conversely, positive contributions indicate a positive association with OPE, thus moving the probability of a higher OPE towards the better-off group [32]. Thus, higher education in the years 2007, 2010 and 2011 and higher income in the years 2009, 2011 and 2013 were associated with a lower risk of higher OPE for the worse-offs. A positive contribution of age in the years 2005, 2006, 2007, 2014 and 2016 indicated that the risk of higher OPE decreased with age. For almost all the years (except for 2012), the effect of regions was substantially great. Intuitively, a positive contribution of settlement of residence in the years 2007, 2009, 2010, 2013 and 2016 indicated that residing in cities and towns statistically draws higher OPE towards the better-off group compared with the better-offs from the small urban settlement and village population. However, the differential approach of decomposing CI leaves a large part unexplained (random effect residuals) in particular for 2007, 2009 and 2014. The large part of such approximation error could be assigned to regions (the size of residuals for the year became smaller on decomposition for each of 39 regions representing the study sample; results were too exhaustive to present here).

**Table 3:** Concentration index and decomposition of concentration indices

	2016	2015	2014	2013	2012	2011	2010	2009	2007	2006	2005
CI	0.028	0.018	0.004	0.011	0.020	0.014	0.031	0.025	0.024	0.027	0.035
*Contribution (%)*											
Age	0.337	− 6.144	8.152	− 0.931	− 0.334	− 0.019	− 2.562	− 1.554	0.114	1.913	3.295
Education	0.00	17.15	71.33	19.90	1.19	− 0.13	− 2.80	9.52	− 3.00	0.17	0.00
Income	26.65	63.40	333.03	− 79.77	76.02	− 42.18	38.15	− 109.79	35.86	24.09	108.40
Settlement of residence	15.48	− 25.21	− 36.93	59.09	− 9.60	− 33.51	4.05	3.78	23.27	− 1.91	− 14.21
Household size	− 0.98	3.69	− 37.89	2.08	− 1.11	7.40	0.55	0.18	1.59	1.70	6.04
Fixed effects (Regions)	7.32	69.32	209.63	126.70	− 2.24	117.61	58.51	21.05	187.88	68.01	7.02
Residuals	51.20	− 22.21	− 447.32	− 26.95	36.07	51.21	4.10	176.81	− 145.72	6.02	− 10.54
Total	100	100	100	100	100	100	100	100	100	100	100

Using RIF regression decomposition, we examined the difference in the 10th, 50th (median) and 90th quantiles of OPE distribution. A positive CI at the 90th quantile of OPE for all the years except 2005, and at median OPE, except for 2012, indicated a probability of higher OPE towards better-offs in top-end OPE, barring the years 2005 and 2012. In almost all the years (except 2005 and 2012), a higher OPE was associated with a higher level of education for the better-offs in the 90th quantiles of OPE. A negative contribution of SEP at the median OPE implied that an increasing affluence level decreased the risk of higher OPE for the worse-offs in 2007, 2010, 2012 and 2013. The negative contribution of settlement of residence reflected the fact that residing in cities and towns pushed the worse-offs towards a higher OPE in the 10th quantile of OPE in 2016; in the 50th quantile of OPE in 2006, 2011 and 2013; and in the 90th quantile of OPE in 2006 and 2016. The negative contribution of household size suggested a concentration of large households among the worse-off population had lowered the risk of higher OPE for the worse-offs in the 10th quantile of OPE in 2005, 2013 and 2016; in the 50th quantile, in 2011; and in the 90th quantile, in 2005. Much of the random effect residuals for the 90th quantile of OPE in 2006 and in 2016, and for the 10th quantile of OPE in 2016 could be assigned to settlement of residence (Table 4).

**Table 4:** Concentration index and decomposition at the mean for different quantiles of OPE

	2016			2015			2014			2013		
Centiles (OPE) ➝	10th	50th	90th	10th	50th	90th	10th	50th	90th	10th	50th	90th
CI	− 0.001	0.037	0.006	0.004	0.037	0.292	0.011	0.041	0.214	− 0.008	0.014	0.214
*Contribution (%)*												
Age	0.007	− 0.001	− 0.054	− 0.508	− 0.511	− 0.242	− 0.479	− 0.261	− 0.241	0.748	− 0.898	− 0.078
Education (3 = highest)	446.23	38.86	528.87	51.13	− 27.20	46.04	13.33	16.37	14.86	− 123.27	438.70	17.85
SEP (5 = poorest)	745.16	7.65	253.83	− 13.54	14.73	1.87	− 2.24	1.00	− 0.81	− 56.74	− 221.66	1.93
Settment of residence	− 1002.12	47.53	− 875.65	46.12	102.47	46.84	83.16	75.08	79.12	299.78	− 162.59	76.76
Household size	− 34.61	2.16	111.62	10.14	5.57	3.41	0.89	2.01	4.34	− 9.46	23.19	1.81
Residual	− 54.67	3.80	81.38	6.65	4.93	2.09	5.34	5.79	2.73	− 11.05	23.26	1.73
Total	100	100	100	100	100	100	100	100	100	100	100	100

	2012			2011			2010			2009		
Centiles (OPE) ➝	10th	50th	90th	10th	50th	90th	10th	50th	90th	10th	50th	90th
CI	0.013	− 0.103	0.070	0.013	− 0.011	0.026	0.010	0.045	0.373	0.008	0.025	0.390
*Contribution (%)*												
Age	− 0.438	0.139	− 0.518	− 0.011	0.039	− 0.041	− 0.000	− 0.000	− 0.000	− 0.001	− 0.000	0.000
Education (3 = highest)	25.26	− 22.92	− 24.80	− 9.31	230.18	52.15	119.36	18.22	2.07	9.78	23.35	12.14
SEP (5 = poorest)	− 47.76	− 22.73	45.42	6.36	123.57	− 248.14	− 84.84	− 5.10	1.67	− 107.06	15.25	7.16
Settment of residence	113.23	153.33	55.31	78.00	− 167.91	221.49	26.16	78.76	90.12	173.78	50.42	76.86
Household size	1.30	− 3.55	14.76	10.99	− 40.30	38.99	15.88	3.11	3.51	10.65	4.62	2.27
Residual	8.40	− 4.27	9.83	13.97	− 45.58	35.55	23.44	5.01	2.63	12.86	6.36	1.56
Total	100	100	100	100	100	100	100	100	100	100	100	100

	2007			2006			2005		
Centiles (OPE) ➝	10th	50th	90th	10th	50th	90th	10th	50th	90th
CI	0.060	0.108	0.068	0.004	0.009	0.001	0.021	0.023	− 0.021
*Contribution (%)*									
Age	0.000	− 0.000	− 0.000	− 0.005	− 0.003	− 0.044	− 0.002	− 0.001	0.003
Education (3 = highest)	15.57	22.26	45.05	− 140.17	14.94	5883.82	− 60.23	− 5.10	− 486.75
SEP (5 = poorest)	5.13	− 8.07	23.88	2.88	117.09	2875.00	22.13	19.04	− 176.17
Settment of residence	74.87	81.09	2.54	136.18	− 95.37	− 10,502.91	132.75	64.60	881.77
Household size	0.65	1.04	13.76	39.66	24.87	929.19	− 0.28	5.38	− 61.92
Residual	3.77	3.68	14.78	61.45	38.47	914.95	5.63	16.08	− 56.94
Total	100	100	100	100	100	100	100	100	100

Table 5 shows the effect of covariates on different quantiles of OPE for the richest and the poorest population. The decomposition analysis indicated that, at the 90th quantile of OPE, living in an urban settlement made a significant positive contribution to the total structural effect for the richest in 2009. At the median, living in a town made a significant negative contribution in 2007, 2012 and 2014, as it did for the richest living in a rural settlement in 2016. At the median, living in an urban settlement had a significant positive contribution in 2007, 2009 and 2010, as did living in a rural settlement in 2006 for the richest. At the median, living in a town had a significant negative contribution in 2005 and 2012, whereas living in a rural settlement had a significant positive contribution in 2012 for the poorest (Table 5).

**Table 5:** RIF–OLS estimates of covariates on OPE

Variables	Centile-OPE	2016		2015		2014		2013		2012		2011	
		Richest	Poorest	Richest	Poorest	Richest	Poorest	Richest	Poorest	Richest	Poorest	Richest	Poorest
Age	10th	0.01	− 0.01	0.00	0.01	− 0.01	− 0.03	− 0.01	0.02	0.01	− 0.02	− 0.02	− 0.01
	50th	0.01	− 0.01	− 0.01	0.00	0.01	− 0.02	0.00	0.02	0.01	− 0.04**	− 0.01	0.00
	90th	0.03*	0.02	0.00	− 0.01	0.00	0.00	− 0.03	0.05*	0.00	− 0.02	− 0.01	0.01
*Education (comparison group: below secondary school)*													
Completed secondary school	10th	− 0.41	1.01	− 0.41	− 0.59	− 0.60	− 0.16	− 0.47	− 0.92	0.55	1.03*	− 0.29	− 0.33
	50th	− 0.31	− 0.38	0.40	− 0.14	0.84	− 1.13	0.07	− 0.55	− 0.40	0.23	− 0.47	0.19
	90th	1.17	− 0.16	1.09	− 1.37	0.48	1.96	0.33	− 3.53*	− 0.53	1.76	− 0.63	0.33
Higher education incl. vocational training	10th	− 0.67	0.83	− 0.70*	0.21	− 0.28	0.82	− 0.57*	− 0.03	− 0.07	0.18	− 0.67	0.29
	50th	− 0.28	− 0.30	0.31	0.63	0.68	0.10	− 0.52	− 0.51	− 0.13	− 0.50	0.28	0.31
	90th	0.67	1.03	− 0.30	− 1.34	0.90*	0.95	1.14	− 3.02	0.08	1.09	0.21	0.22
*Settlement of residence (comparison group: Oblast city)*													
Town	10th	− 0.33	− 1.42	0.54*	0.62	− 0.15	− 1.24	0.00	0.41	− 0.67	− 0.83	0.13	0.11
	50th	− 1.02	− 0.53	0.58	− 0.47	− 2.29***	− 0.49	− 1.01*	− 0.25	− 1.60***	− 3.05***	− 0.35	0.63
	90th	− 0.44	− 1.90	0.13	− 0.15	− 0.28	0.71	− 0.58	0.55	− 0.37	− 0.78	− 0.46	0.20
Small urban settlement	10th	0.63	− 0.01	− 1.93	0.00	− 2.33	0.30	0.62	− 0.93	− 1.20	− 1.82	− 1.92	0.47
	50th	− 1.17	− 0.54	0.99	0.00	− 0.64	− 0.16	1.11	− 0.31	− 0.55	− 0.72	− 0.94	0.84
	90th	− 0.67	− 2.21	− 0.51	0.00	− 1.00	1.72	− 0.14	− 0.26	− 0.46	0.81	1.42	0.49
Rural	10th	− 1.55	0.15	− 0.98	0.17	0.86	− 0.47	− 0.50	0.71	0.30	− 0.28	− 1.91	− 0.65*
	50th	− 2.02***	0.22	− 0.90*	− 0.70	− 0.45	0.12	− 0.51	0.63	− 0.44	− 1.61**	− 1.12	− 0.64
	90th	− 0.22	− 0.10	1.06	− 0.23	2.49	1.04	− 1.40*	− 0.63	− 0.30	− 0.97	− 0.94**	− 0.93
Household size	10th	0.08	− 0.15	0.35	0.22	− 0.45*	− 0.12	0.03	0.13	0.05	− 0.33*	− 0.34	0.07
	50th	0.13	− 0.04	0.55***	0.14	− 0.15	− 0.18	− 0.10	0.10	0.15	− 0.19	− 0.03	0.07
	90th	0.02	− 0.10	0.52	− 0.08	− 0.04	− 0.11	0.15	0.08	0.31	− 0.19	0.15	− 0.02
Constant	10th	6.18***	7.10***	5.99***	4.93***	8.08***	8.26***	7.26***	4.54***	5.38***	8.06***	7.51***	6.47***
	50th	7.85***	8.55***	6.29***	6.77***	7.57***	9.72***	8.02***	6.56***	7.01***	11.41***	7.90***	6.90***
	90th	7.86***	8.65**	7.69***	10.25***	8.91***	7.67***	10.26***	9.33***	8.32***	10.37***	9.09***	8.37***
*N*		*14,913*		*14,717*		*14,786*		*17,516*		*18,183*		*17,843*	

Variables	Centile - OPE	2010		2009		2007		2006		2005	
		Richest	Poorest	Richest	Poorest	Richest	Poorest	Richest	Poorest	Richest	Poorest
Age	10th	0.00	0.01	0.02	− 0.01	0.00	0.01	0.01	0.00	− 0.03*	− 0.01
	50th	0.01	0.00	0.01	0.00	− 0.01	0.00	0.00	− 0.01	− 0.01	0.00
	90th	0.01	− 0.02	0.04*	0.05	0.01	− 0.02	− 0.01	0.02	− 0.01	0.02
*Education (comparison group: below secondary school)*											
Completed secondary school	10th	0.17	0.07	1.54	− 1.56	− 0.36	0.12	− 0.01	0.04	0.98	0.99
	50th	0.22	− 0.19	1.16	0.84	− 0.11	− 0.81	0.58	0.36	0.36	− 0.44
	90th	− 0.07	− 1.48	0.22	1.21	0.52	0.29	− 0.05	0.83	0.81	1.14
Higher education incl. vocational training	10th	− 0.15	0.00	1.84	− 0.59	0.18	0.47	0.46	0.24	1.06	1.14
	50th	− 0.03	− 0.33	1.04	0.52	− 0.27	− 0.41	0.48	0.29	0.39	− 0.04
	90th	0.31	− 1.49	0.40	0.82	0.29	0.81	0.14	0.86	1.86*	0.35
*Settlement of residence (comparison group: Oblast city)*											
Town	10th	− 0.23	− 0.52	− 0.42	0.01	− 0.72	− 0.06	− 0.21	− 0.26	− 0.23	− 0.88
	50th	− 0.47	− 1.69*	− 0.61	− 0.45	− 1.04**	0.25	− 0.66*	− 0.25	− 0.66	− 2.49***
	90th	0.23	1.60	− 0.46	− 0.38	− 0.58	− 0.48	− 0.45	− 0.05	− 0.95	− 0.45
Small urban settlement	10th	0.27	0.04	− 0.33	0.22	− 0.11	0.05	0.53*	− 0.06	− 1.84	0.41
	50th	1.24***	0.12	1.09**	− 1.27	1.48***	− 0.85	− 0.81	1.33*	0.33	− 0.79
	90th	2.22	2.13	4.96***	− 2.03	− 0.90*	− 1.42	− 0.72**	− 0.16	− 2.40**	0.51
Rural	10th	0.43*	− 0.37	1.38	− 0.78	− 0.60	− 0.68	0.22	− 0.54	− 2.04*	− 0.48
	50th	− 0.29	− 0.86	− 1.42	− 0.77	− 0.61	− 0.29	1.21***	− 0.07	− 1.33*	− 1.15
	90th	− 0.51*	0.04	0.36	− 1.35	− 0.91*	− 0.97	− 0.98**	0.02	− 1.07	0.08
Household size	10th	0.01	0.02	0.07	0.11	0.08	0.00	− 0.01	0.19	− 0.17	0.01
	50th	0.09	0.12	− 0.15	− 0.03	− 0.27*	− 0.24*	0.21	0.07	0.09	− 0.02
	90th	− 0.06	0.15	0.66	0.10	− 0.03	− 0.17	− 0.28	0.15	− 0.53	− 0.05
Constant	10th	5.37***	5.73***	2.81*	6.36***	5.00***	4.30***	4.47***	4.14***	5.86***	4.16***
	50th	6.64***	7.69***	6.05***	6.82***	7.87***	7.46***	5.83***	5.76***	5.99***	7.16***
	90th	7.88***	9.91**	4.73***	5.79**	7.25***	9.70***	9.08***	5.29***	8.58***	6.19**
*N*		*17,363*		*11,489*		*11,939*		*12,082*		*9964*	

***p < 0.001, **p < 0.01, *p < 0.05

A statistically highly significant constant for all quantiles of OPE in all the years indicated that mean value of OPE was significantly different from zero when other covariates were set to zero. The intercept coefficients suggested the highest expected mean value of OPE was for the poorest at the 10th quantile of OPE in 2014 and at median and at the 90th quantile of OPE, in 2012. Although not consistently, we could find that the settlement of residence had some effect on different quantiles of OPE for the richest and for the poorest in some years; however, the effect of other covariates on different quantiles of OPE were of little significance (Table 5).

Table 6 presents the effects of different covariates on different quantiles of OPE for urban and rural population. At the median and at the 90th quantile, higher education and, at the 10th quantile, the poorest SEP made significant positive contributions in 2011 to the total structural effect for the urban population. At the 90th quantile, the second-poorest SEP in 2013 and the poorest SEP in 2014 made a significant negative contribution to the total structural effect for the urban population. Such observations supported the assumption that awareness impinged upon higher education and deprivation in the purportedly unconstrained supply situation for urban locations had driven the probability of higher OPE. At the 90th quantile, the second-richest SEP in 2015 and the middle SEP in 2009 and, at the median, the second-richest and middle SEPs in 2015 made a significant positive contribution to the total structural effect for the rural population.

**Table 6:** RIF–OLS estimates of covariates on OPE

Variables	Centile-OPE	2016		2015		2014		2013		2012		2011	
		Urban	Rural	Urban	Rural	Urban	Rural	Urban	Rural	Urban	Rural	Urban	Rural
Age	10th	0.01	0.00	0.00	− 0.02	− 0.01	− 0.02	− 0.01	− 0.01	0.00	− 0.05**	− 0.01*	− 0.01
	50th	0.01*	0.02	0.00	− 0.01	− 0.01	0.00	0.00	− 0.02	0.00	− 0.01	− 0.01	− 0.02
	90th	0.01	0.04	0.01	− 0.02	0.01	0.01	0.00	− 0.01	0.00	0.00	0.00	− 0.01
*Education (comparison group: below secondary school)*													
Completed secondary school	10th	0.14	0.53	− 0.04	− 0.68	0.54	0.23	− 0.22	0.73	0.19	0.09	0.38	− 0.48
	50th	− 0.14	0.25	− 0.11	− 0.38	0.64	− 0.06	− 0.02	0.3	− 0.39	− 0.07	0.22	− 0.58
	90th	0.46	− 0.1	0.31	− 0.28	0.15	2.84	0.64	− 0.75	0.05	0.39	0.56*	− 0.48
Higher education, incl. vocational training	10th	0.35	0.08	− 0.17	− 0.33	0.64	1.06	0.03	− 0.02	− 0.03	0.13	0.51	− 0.04
	50th	− 0.3	0.48	0.22	− 0.1	0.90*	0.58	− 0.19	− 0.62	− 0.05	− 0.12	0.85***	− 0.98
	90th	0.12	0.13	0.25	− 0.46	0.91*	1.06	0.3	− 0.99	0.68*	0.72	0.91***	0.50
*SEP (comparison group: Richest quintile)*													
Second-richest	10th	0.23	− 0.16	− 0.2	1.7	0.22	0.35	0.15	0.59	− 0.03	− 0.67	− 0.03	1.39
	50th	− 0.41	1.09	− 0.13	3.02***	− 0.08	0.61	0.1	0.73	0.12	− 0.16	− 0.12	0.52
	90th	− 0.94	1.51	0.05	6.54***	0.13	2.7	− 0.88	1.39	0.57	0.07	0.19	0.19
Middle	10th	0.14	0.67	− 0.05	1.53	0.2	− 1.76	0.02	0.74	− 0.06	0.25	0.19	1.75
	50th	− 0.71*	0.1	− 0.21	2.91***	− 0.21	− 0.77	0.14	1.1	− 0.26	0.25	0.15	1.51
	90th	− 1.15*	2.27	0.06	2.78*	− 0.57	1.83	− 1.02*	0.73	0.00	0.52	0.27	1.08
Second-poorest	10th	− 0.11	0.01	0.04	1.28	0.06	0.65	− 0.19	0.52	− 0.11	0.14	0.21	1.09
	50th	− 0.68	− 0.74	− 0.11	1.70*	− 0.32	0.18	− 0.39	0.11	− 0.49	0.25	0.14	0.17
	90th	− 0.75	− 0.66	− 0.11	0.64	0.36	0.81	− 1.73***	0.59	− 0.09	0.27	0.72	− 0.12
Poorest	10th	0.08	0.44	− 0.04	1.21	− 0.06	0.16	− 0.71	0.91	− 0.25	0.49	0.60**	1.44
	50th	− 0.80*	0.84	0.01	0.39	− 0.13	0.49	− 0.3	− 0.21	− 0.28	− 0.08	0.19	0.08
	90th	− 0.78	0.07	− 0.3	0.08	− 1.22**	1.33	− 1.35*	0.68	0.19	− 0.06	− 0.12	− 0.11
Household size	10th	0.04	− 0.01	0.05	0.08	− 0.07	− 0.12	0.04	− 0.13	0.09	− 0.34*	0.05	0.06
	50th	0.22**	0.18*	0.16	− 0.05	− 0.08	− 0.19	0.1	− 0.11	0.13	− 0.1	0.12	− 0.01
	90th	0.15	0.18	0.12	− 0.05	0.11	− 0.02	0.24	− 0.13	0.03	0.22	0.12	− 0.02
Constant	10th	5.66***	6.14***	6.41***	5.92***	6.13***	6.94***	6.21***	6.34*	5.54***	8.72***	5.67***	4.74***
	50th	7.35***	5.22***	7.09***	7.25***	7.37***	7.75***	7.27***	9.12***	6.99***	7.83***	6.49***	8.23***
	90th	9.16***	6.37***	8.10***	9.91***	7.89***	7.01***	9.13***	9.86***	8.54***	7.55***	7.78***	9.15***
*N*		*14,913*		*14,717*		*14,786*		*17,516*		*18,183*		*17,843*	

Variables	Centile-OPE	2010		2009		2007		2006		2005	
		Urban	Rural	Urban	Rural	Urban	Rural	Urban	Rural	Urban	Rural
Age	10th	0.00	− 0.01	0.00	− 0.01	− 0.01*	0.01	− 0.02**	0.00	0.00	− 0.01
	50th	− 0.01	0.00	0.00	0.00	− 0.01	0.00	− 0.01	0.01	0.01	− 0.01
	90th	− 0.01	0.02	0.02*	− 0.03*	0.00	− 0.01	− 0.01	− 0.01	0.01	0.00
*Education (comparison group: below secondary school)*											
Completed secondary school	10th	0.07	0.1	0.46	− 0.08	− 0.3	0.01	− 0.49	− 0.11	0.24	1.43
	50th	0.06	0.3	0.46	0.13	0.07	− 0.56	0.15	0.7	0.2	− 0.37
	90th	− 0.3	0.64	− 0.5	0.08	0.58*	0.87	0.1	0.43	0.51	− 0.16
Higher education, incl. vocational training	10th	0.25	0.01	0.67	0.2	0.21	0.31	0.3	− 0.55	0.3	1.74*
	50th	0.19	0.29	0.75*	0.28	0.38	− 0.22	0.22	0.86	0.2	0.31
	90th	0.30	0.27	− 0.69	0.72	0.57**	0.98	0.27	0.61	0.6	− 0.19
*SEP (comparison group: Richest quintile)*											
Second-richest	10th	0.3	− 0.37	− 0.06	0.06	− 0.18	0.16	− 0.33	− 0.42	− 0.72**	2.18
	50th	0.23	− 0.5	− 0.31	0.27	0.09	− 0.46	− 0.25	− 0.07	− 0.21	0.11
	90th	0.58	− 0.24	− 0.14	1.6	− 0.25	1.34	− 0.06	0.96	− 0.82*	− 0.13
Middle	10th	0.49*	− 0.58	0.70*	0.07	− 0.03	0.52	− 0.04	− 0.03	− 0.12	1.73
	50th	− 0.23	− 1.15	0.3	2.33*	0.16	0.79	0.1	− 0.8	− 0.46	0.58
	90th	0.59	− 0.81	0.07	3.22**	0.11	2.22	− 0.32	0.22	− 0.29	0.37
Second-poorest	10th	0.28	− 0.25	− 0.48	− 0.5	0.03	0.46	− 0.17	− 0.73*	− 0.26	1.86
	50th	− 0.07	− 0.51	− 0.22	0.5	− 0.02	− 1.46*	− 0.03	− 0.67	− 0.25	0.08
	90th	− 0.02	0.39	− 0.59	1.03	0.18	0.58	0.13	1.21*	− 0.43	− 0.77
Poorest	10th	0.47	− 0.19	0.75*	− 0.35	0.11	0.08	− 0.56	− 0.69	− 0.52	1.96
	50th	− 0.42	0.07	0.08	0.52	0.06	− 0.59	− 0.85**	− 0.42	− 0.71	0.57
	90th	0.89	0.51	1.01	1.45*	0.26	0.46	− 0.43	0.69*	− 0.89	0.38
Household size	10th	0.01	− 0.05	− 0.02	0.01	− 0.07	0.07	− 0.07	0.06	0.06	0.05
	50th	0.06	0.07	0.05	− 0.12	0.05	− 0.17	0.15*	0.07	0.21**	− 0.1
	90th	0.14	0.11	0.30*	− 0.40*	0.11	− 0.04	− 0.08	− 0.06	0.09	0.02
Constant	10th	5.35***	6.44***	4.41***	6.17***	5.69***	3.70**	5.94***	5.10***	4.43***	1.78
	50th	6.99***	6.53***	6.12***	6.10***	6.27***	7.18***	6.05***	5.19***	4.92***	5.90***
	90th	8.09***	6.78***	7.15***	9.28***	6.99***	6.79***	8.25***	6.64***	6.99***	7.33***
*N*		*17,363*		*11,489*		*11,939*		*12,082*		*9964*	

***p < 0.001, **p < 0.01, *p < 0.05

Further, Tables 5 and 6 showed that the structural effect of household size was significantly positive at the median OPE for the richest in 2015 and for the urban population in 2016. Household size in 2012 and living in a rural settlement in 2011 had a negative effect on OPE at the 10th quantile of OPE for the poorest. Taken together, these findings suggested that household size shifted the probability of higher OPE for the richest and urban population.

The highly significant intercept coefficients suggested that the highest expected mean value of OPE was for the rural population at the 10th quantile of OPE and at the median, in 2012; and at the 90th quantile of OPE in 2015 (Table 6).

## Discussion

This study addressed differential impacts of socioeconomic position and of the urban–rural segmentation of population in the distribution of OPE. We also examined changes in the distribution of OPE between the worse-off and the better-off Russians over a 12 year (2005–2016) period.

A substantially high mean OPE compared with the median (Table 2) indicated a considerable inequality in OPE distribution. We found (a Kruskal–Wallis H test was conducted) that there was a statistically significant difference in the median OPE between the three different groups of the independent variable, level of education. This finding supports the relationship between level of education and visit frequency to a doctor (Table 2) and subscribes to the findings from earlier studies about the effect of education attainment on household healthcare expenditure.

The inflation-adjusted income gap between the richest and the poorest were at its peak (98%) in 2006^14^ but we found no difference in mean OPE for the richest and the poorest quintiles at any quantile of OPE, however, denial of access to healthcare services attributable to financial affordability was higher among the better-offs. This quirky revelation from this study is well aligned with the financing mechanisms of the health system in a hierarchical social structure where in one hand, service provision is constrained by rationing from the compulsory insurance fund and at the same time discretion of the service providers (doctors and nurses) determine service availability while on the other, individual behaviour in public realm influences position and acceptance of the individual in the society. This finding is an add on contribution to the earlier studies of Balabanova et al. (2012) and Eaddy et al. (2012). Such observation guides policy response for (1) defining boundaries between free and chargeable health that is often blurred, (2) eliminating variances in substitution between formal and informal payments across regions, and (3) addressing gap between population entitlement and available provision of healthcare services.

Notwithstanding inconsistency in the direction of the distribution effect over the study period, the mean-based (Blinder 1973; Oaxaca 1973) decomposition of the CI indicated income in 2005, 2009, 2012, 2013, 2014, 2015 and 2016, followed by education in 2013, 2014 and 2015 and settlement of residence in 2013, 2014 and 2016, were the most important contributor(s) to OPE distribution. The contribution of age followed our sample characteristics. Interestingly, the risk of higher OPE rose with increasing age in 2009, 2010 and 2015, contrary to the risk of higher OPE, which decreased with increasing age in 2005, 2006 and 2014. When percentage of elderly (75 years and above) female respondents were declining consistently, and respondents with reported ill health were relatively less in 2009, 2010 and 2015 compared to other years (Table 2), such observations indicated that risk of higher OPE in these years were attributable to gender effect and reported ill health was not necessarily the only determinants of OPE. The region effect on OPE distribution was substantial in all the years except in 2005, 2012 and 2016. The positive contribution of education implying higher OPE for the better-offs was a consequence of the conventional relationship between affluence and education.

We examined effect of different covariates on 10th, 50th (median) and 90th quantiles of OPE distribution for different groups of population. Although mean OPE increased substantially across SES during the study period, a negative shift of the CI by 20% indicated an overall concentration of OPE amongst the better-off group of Russians between 2005 and 2016. However, this change is not the same in different quantiles of OPE. The CI of OPE at the 10th quantile of OPE decreased by 106 + percent and at 90th quantile by 130%, but the CI of OPE at the 50th quantile (median) of OPE increased by 60%. Such reflection directs targeted policy response for the worse-off at median OPE, ceteris paribus.

We decomposed the OPE gap between the richest and the poorest, and between urban (oblast city and town) and rural (small urban settlement and village) population into structural and composition effects. With our objectively determined SEP, we could not find a significant distributional difference in mean OPE at any quantile of OPE between the richest and the poorest quintiles of SES in the study period. However, the statistically significant effects of urban and rural settlements on OPE at the median could be attributed to a combination of incurred OPE by a relatively higher proportion of respondents in urban settlements (namely, respondents residing in oblast cities and towns) and the prevalence of a relatively higher level of OPE in urban settlements (Table 2). Albeit without any definite order in the difference between mean OPE for urban and rural population (namely, respondents residing in small urban settlements and villages), we may ascribe such temporal variability (that we found in 2006, 2007, 2010, 2013 and 2016) to urban-rural distribution of respondents (Table 2). Further, such revelation calls for examining pattern of service consumption for urban and rural population (that is beyond the scope of this study) before drawing any policy conclusions.

Compared to living in an oblast city, living in an urban settlement made an overall significant positive contribution to the total structural effect at the median and at the 90th quantile of OPE for the richest, whereas living in a town made an overall significant negative contribution at the median of OPE for the richest and the poorest. Thus, at the median and top end of the OPE effect on the level of affluence dominated over the settlement of residence. Such temporal variations in urban–rural differential impact can be explained by considerable turbulence in living standards at any given level of living in the Russian Federation, as has been observed by Lokshin and Ravallion (2000).

Notwithstanding the temporal variations and variability in the direction of effects, it became apparent that structural effects of SEP and settlement of residence compensated for differences in observable composition effects at the median OPE distribution. Different quantiles were having different composition effects. Composition effects were predominantly driven by differences in the distribution of educational level, SEP, and settlement of residence; the effects of a large household size were present for few years. As was evident at the mean-based decomposition, region effect was substantial at every quantile in most of the years. The structural effects were observed preponderantly at the median and at the 90th quantile of OPE. Thus the inequality in OPE was not due to difference in OPE at the low-end of distribution, and was driven by the differences in structural effects.

In summary, urban settlement contributed to the top end of OPE distribution for the richest and town settlement contributed at the median for the richest and the poorest. The SEP effect was absent at the top end of OPE and the effects of settlement of residence were observed at the low end of OPE in general. Considering the right-skewed distribution of OPE in our data, an increased CI of OPE at the median suggested an increased burden of OPE for the worse-offs over the time. Such findings are congruent with Baird’s (2016) observations. Finally, the effect(s) of covariates on OPE were quite unstable for the reasons attributable to a significantly high subjective component (random effect residuals) inherent in the system. Such subjective components can be ascribed to the decentralised characteristics of service provisioning with year-on-year fluctuations in financing that is centralised, the federal compulsory health insurance fund. Such phenomena are the result of an economy that is highly vulnerable to the currency fluctuations in the external market, and that is also susceptible^15^ to export fluctuations.

## Conclusion

OPE inequality has received little attention in health economics. This study sheds light on the plausible factors contributing to OPE differences (inequality) in the richest and the poorest groups and in the urban and the rural population of the Russian Federation. Differences in OPE between groups were decomposed into two parts: one explained by differences in the distribution of characteristics (composition effect) and one explained by differences in the impact of these characteristics (structural effect). The composition effect accounts for a large share of the total differences between the richest and the poorest groups and in the urban and rural population at the mean and at different quantiles of OPE. Conditional on having similar characteristics, there are differential impacts of these characteristics (structural effects) between the urban and the rural population. Furthermore, unobserved regional effects are important in explaining OPE differences between the richest and the poorest groups and between the urban and the rural population in the Russian Federation.

Our methodological approach unmasked the effect of covariates at different quantiles of OPE rather than portraying average effects that often hide the relationship between explanatory variables and outcomes elsewhere in the distribution (Binder and Coad, 2011). The effect of sample variability that is inherent in multiple waves of a longitudinal survey over a long period of time cannot not be ignored, however, the use of a large sample size has reduced the level of uncertainty in our analysis. The population who could not afford OPE are not accounted for in our study, and a selection process prevails; therefore, an underestimated structural effect cannot be ruled out. The reference group chosen influences the measure of composition effect and structural effect, so the results are highly contextualised. The use of categorical variables (education, SEP and settlement of residence) limits a comparison of results across studies unless the choice of omitted category remains the same.

Although our methodological approach cannot have a causal interpretation, our results present evidence that calls for complex policy interventions to address OPE inequality at different distributions of OPE for different SEPs and for different settlements of residence in the Russian Federation. To conclude, this study establishes the need to unfold (1) the inflationary effect of OPE on household consumption expenditure for different SES, and (2) the differential effects of federal compulsory insurance fund on health facility expenses in different regions of the Russian Federation.

## Footnotes

1 This measure provides an indicator of the degree to which health policy exposes citizens to a substantial financial risk associated with needed healthcare services (Cunningham PJ. Chronic Burdens: The Persistently High OOP Health Care Expenses Faced by Many Americans with Chronic Conditions. Commonwealth Fund 2009; New York. [http://www.commonwealthfund.org/publications/issue-briefs/2009/jul/chronic-burdens-the-persistentlyhigh-out-of-pocket-health-care-expenses-faced-by-many-americans]; Collins SR, Rasmussen PW, Doty MM, Beutel S. Too High a Price: Out-of-Pocket Health Care Costs in the United States. Commonwealth Fund November 2014; New York. [http://www.commonwealthfund.org/publications/issue-briefs/2014/nov/out-of-pocket-healthcare-costs]; Schoen CS, Hayes, L, Collins SR et al. 2014. America’s Underinsured. Commonwealth Fund 2014; New York).

2 Compulsory health insurance in The Russian Federation is “universalist”, that is, the entire population outside formal sector employment is exempt from paying contributions.

3 One form of rationing.

4 Payment is based on case-mix adjusted workload of each health facility. The tariff is determined by country-specific clinical disease group and intervention (including surgery) procedures.

5 Because of the decline in response rate in large cities, sample repair was done in Round 15 (2006; http://www.cpc.unc.edu/projects/rlms-hse/data).

6 The RLMS sample compared favourably to those determined by the Soviet census: there is usually a difference of only one percentage point or less between the two distributions.

7 At any given current incomes or expenditures on consumption deflated by standard poverty lines, Ravallion and Loskhin (2001) find evidence that many ‘non income’ factors at the individual and household levels impinge on perceived economic welfare in Russia.

8 There are uncertainties about how best to normalise for heterogeneity in consumption needs such as those stemming from demographic differences between households (Pollak 1991). For example, poverty lines used as deflators may not correctly weight differences in household size or demographic composition.

9 The polychoric correlation coefficient is a measure of association for ordinal variables which rests upon an assumption of an underlying joint continuous distribution.

10 This attempts to maximise the square loadings of the columns.

11 Truncation implies even distribution of SES spread over a narrow range, which makes differentiation between the SES difficult.

12 Recentering allows to identify the intercept and perform Oaxaca-Blinder type decomposition at different quantiles.

13 Running a regression of recentered influence function of the unconditional quantile of the outcome variable (OPE) on the explanatory variables.

14 The gap of such income between the poorest and the richest was the smallest (48%) in 2016 and the highest (98%) in 2006 (Table 2).

15 Russian economy is not an economy with diversified exports and so, insulation from the volatility of global oil and gas prices are not strong enough to sustain committed funding for its health system with continually increasing disease burden.
